# The Exceptional Strong Face-centered Cubic Phase and Semi-coherent Phase Boundary in a Eutectic Dual-phase High Entropy Alloy AlCoCrFeNi

**DOI:** 10.1038/s41598-018-33330-0

**Published:** 2018-10-08

**Authors:** Qiannan Wang, Yiping Lu, Qian Yu, Ze Zhang

**Affiliations:** 1 Center of Electron Microscopy and State Key Laboratory of Silicon Materials, School of Materials Science and Engineering, Zhejiang University, Hangzhou, 310027 China; 2 Key Laboratory of Solidification Control and Digital Preparation Technology (Liaoning Province), School of Materials Science and Engineering, Dalian University of Technology, Dalian, 116024 China

## Abstract

Second phase strengthening has been applied to high entropy alloys (HEAs) for optimizing mechanical properties. In this study, by conducting mechanical testing of a eutectic dual-phase AlCoCrFeNi HEA with homogenous distribution of body-centered cubic (BCC) and face-centered cubic (FCC) lamellar phases inside a transmission electron microscope, we found that although BCC was truly the hard phase, decreasing the proportion of BCC phase in fact increased the strength due to the existence of chemically disordered semi-coherent phase boundaries, which acted as potent impediments to dislocation motion resulting in dense dislocation storage in FCC phases. Moreover, the difficulty in dislocation glide caused massive cross-slip, and the interaction between primary slip arrays and cross-slip systems during deformation increased the rate of dislocation accumulation by forming dislocation substructures, thus making the FCC phases exceptionally strong. Our findings not only revealed the underlying strengthening mechanism of eutectic dual-phase AlCoCrFeNi HEAs, but also shed light on new ways in further optimizing the mechanical properties of HEAs.

## Introduction

Second phase strengthening has long been used to enhance the mechanical performance of commercial alloys, such as ferritic-martensitic steel^1,2^, dual-phase lightweight alloys^3–5^ and single-crystal super alloys^6^. Unlike precipitation strengthening, where precipitated particles of optimum size are prepared to inhibit the dislocation motion and lead to the increase of ultimate strength^7^, dual-phase alloys have nearly equal fraction of two phases. As a result, considerable proportion of phase boundaries are introduced into the alloys, exerting important influence on the deformation behavior and mechanical properties^8,9^. The strengthening effect usually originates from two sides: (1) the combination of the strength of two phases, which can be simply estimated by

$$\bar{\sigma }={f}_{1}{\sigma }_{1}+\,{f}_{2}{\sigma }_{2}$$

where f_1_ and f_2_ are the volume fraction of two phases while σ_1_ and σ_2_ are the strength of two phases; (2) the impediment to dislocation motion due to phase boundaries^10^. Since FCC phases are relatively soft, stronger BCC phases are commonly added to increase the strength^11–13^. Additionally, incoherent phase boundaries normally show significant impediment to dislocation glide while coherent phase boundaries have negligible influence^14^.

Recently, new types of dual-phase high entropy alloys (HEAs) were designed under the inspiration of second phase strengthening, showing good combination of high fracture strength and tensile ductility at room temperature^15,16^. In contrast to the conventional strategies for alloy design, HEAs are developed with at least five metallic elements, emerging as a new frontier of research in recent years^17^. The chemically disordered distributions of multiple elements in HEAs have been suggested to show important influence on dislocation slip^18,19^. In particularly, the high mixture of multiple elements in dual-phase HEAs may form distinctive phase boundary structures compared to conventional ones, which could result in different influence on dislocation behaviors. However, experimental observation and analysis are still lacking for a profound understanding of the deformation mechanisms of dual-phase HEAs.

In this study, a five-component eutectic AlCoCrFeNi HEA, consisting of homogenous distributed body-centered cubic (BCC) and face-centered cubic (FCC) lamellar phases, was prepared by vacuum arc melting and casting. By conducting *in situ* tensile tests inside a transmission electron microscope (TEM), a unique deformation behavior and strengthening mechanism were uncovered. The plastic deformation of the alloy was dominated by dislocation activities in FCC phases, where the frequently observed interactions between the primary and cross-slip systems generated dislocation substructures and partly made the FCC phases exceptionally strong. Importantly, the semi-coherent phase boundaries in this HEA were found to be potent obstructions for dislocation motion, which resulted in ultra-high dislocation density in FCC phases and significant hardening effect. Our findings provide new insights into the second phase strengthening mechanism, which would have significant implications for further improving the mechanical performances of HEAs.

## Results

### The microstructure of AlCoCrFeNi dual-phase high entropy alloy

**Figure 1:**
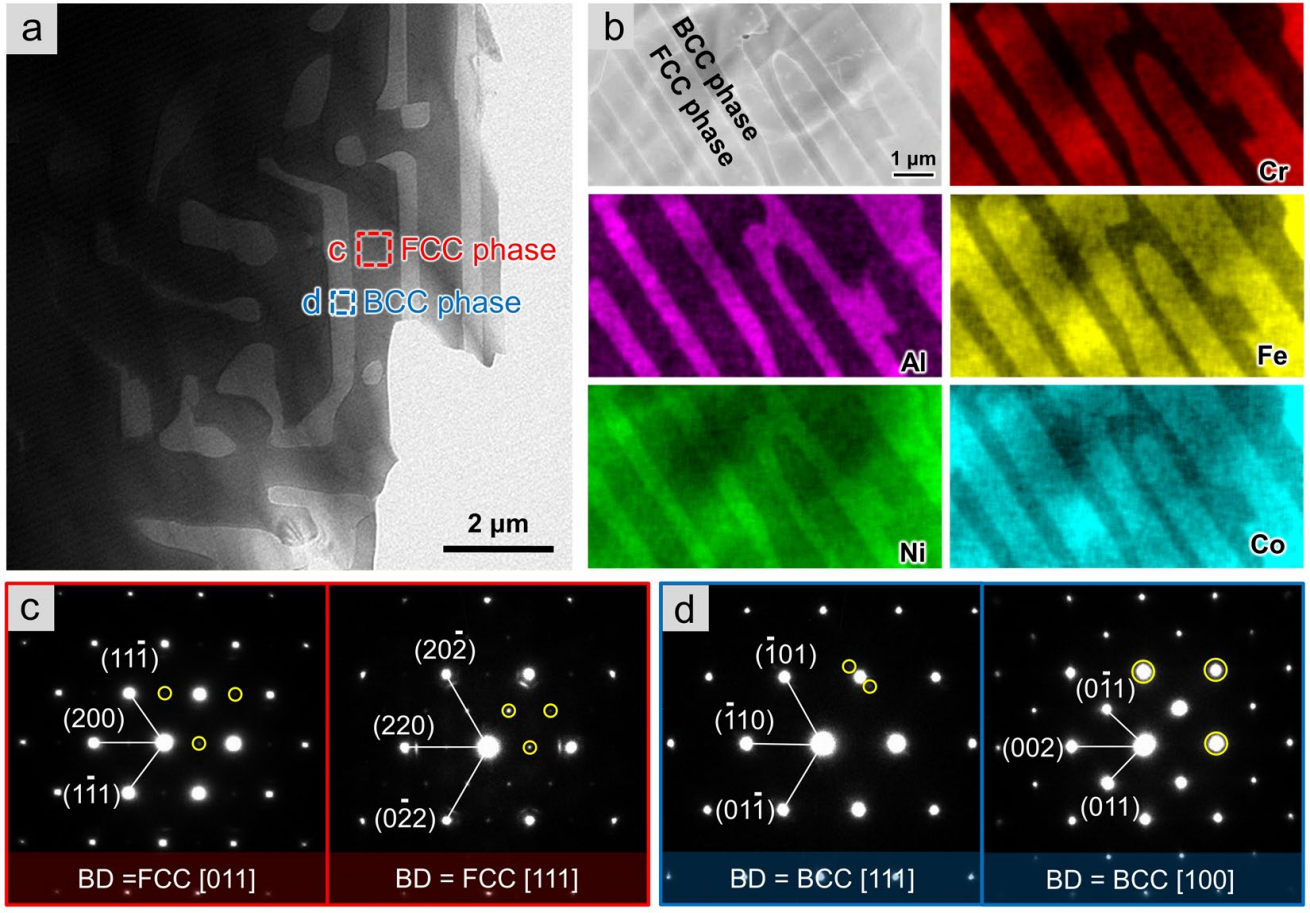
Microstructure and phase constitution of the dual-phase AlCoCrFeNi_2.1_ HEA. (**a**) The TEM image showing the lamellar dual-phase structure and the lamellar thickness was about 1.5 μm for FCC and 0.7 μm for BCC. (**b**) Common but differentiated distribution of elements was characterized by EDS mapping, indicating distinct enrichment of Al and Ni in BCC while Fe, Co and Cr were concentrated in FCC phase. (**c,d**) Diffraction patterns corresponding to the boxed regions in (a) confirmed that this HEA was composed of ordered FCC (L1_2_) and BCC (B2) phase. The superlattice spots were marked with yellow circles.

Figure 1 shows the microstructure and phase constituents of a dual-phase HEA AlCoCrFeNi_2.1_, which demonstrated a uniform and fine lamellar feature, a typical characteristic of eutectic alloys^20–22^. The interlamellar spacing was about 1.5 μm for the larger ones and 0.7 μm for the others. An obvious contrast of the coupled two phases was observed in bright field TEM images (Fig. 1a and S1) owing to the difference in their crystal orientations. Further elemental analysis illustrated that the FCC phase in dark contrast with larger lamellar size was rich in Fe, Co and Cr, while the narrow bright BCC phase was rich in Al and Ni, as shown in Fig. 1b and S2 (Detailed chemical composition was given in Table. S1). Diffraction patterns (Fig. 1c,d) confirmed that the FCC and BCC phases in the AlCoCrFeNi_2.1_ HEA corresponded to the bright thick and dark thin lamellae in Fig. 1a respectively. The condensed interplanar spacing of FCC phase was about 2.06 Å, which was nearly equal to that of the BCC phase of about 2.08 Å. The presence of weak superlattice spots (marked by yellow circles in Fig. 1c,d) in the diffraction patterns suggests that both FCC and BCC phases were ordered structures corresponding to L1_2_ and B2 respectively. Notably, compared to the eutectoid equiatomic AlCoCrFeNi HEAs, the eutectic dual-phase HEAs were characterized by regular lamellar organization with a mixture of BCC and FCC phases, while the former solidified dendritically and generated two BCC phases distinguished by the enrichment of different elements^23^.

### Dislocation dominated plastic deformation in FCC phases

**Figure 2:**
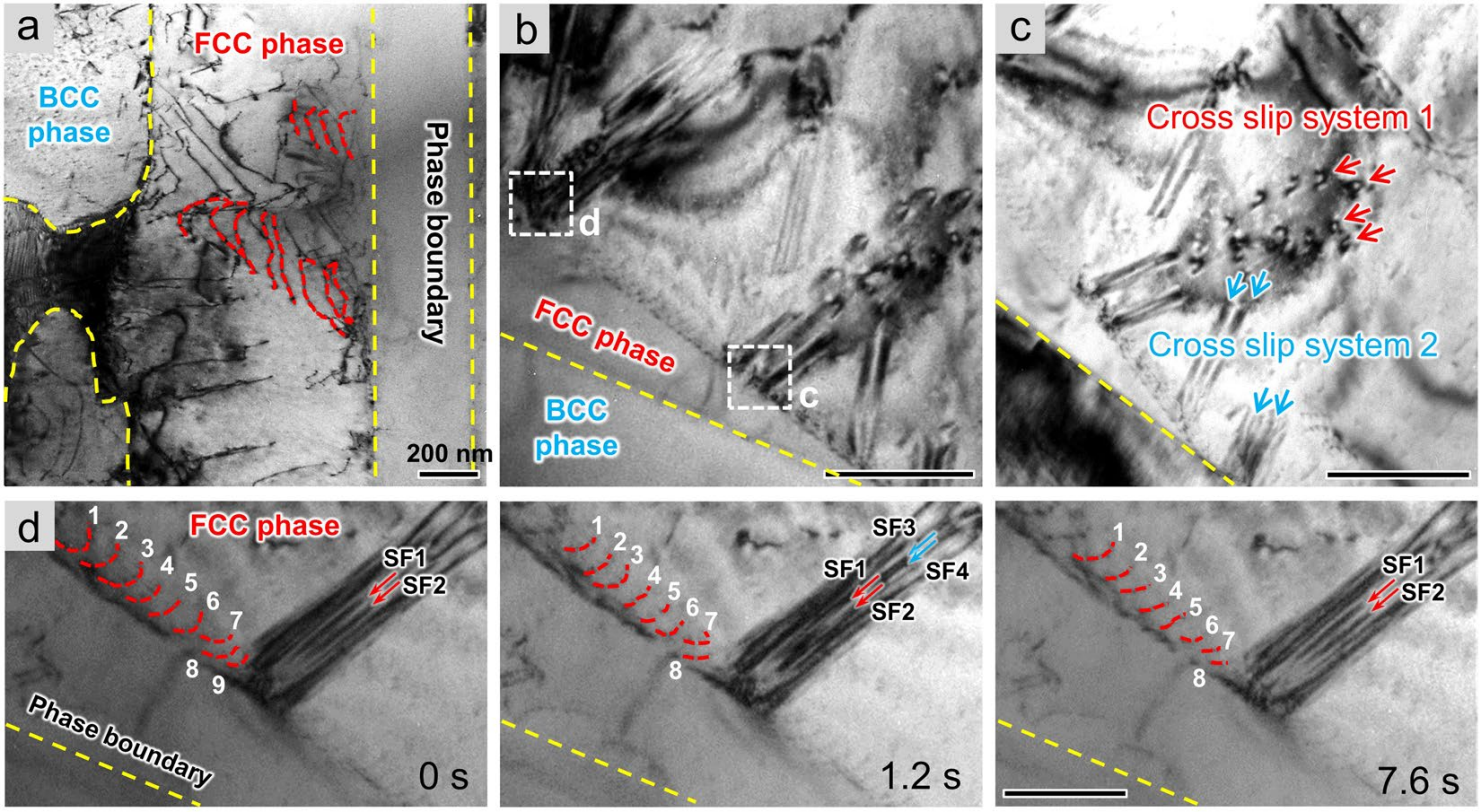
*In situ* deformation behavior of the dual-phase AlCoCrFeNi_2.1_ HEA. (**a**) Plastic deformation of this alloy was dominated by dense dislocation activities in FCC phases where dislocations were emitted from phase boundaries and piled up at the other side, while the BCC counterparts remained undisturbed. (**b**) Cross slip of dislocations in the FCC phase. (**c**) The zoom-in image of boxed region c in (b) showing that there were two cross slip systems activated at the same time. (**d**) Dynamic process of cross slip observed in the boxed region d in (b) illustrated that a full dislocation dissociated into partials and then annihilated at the surface.

To explore the deformation mechanism and the relationship between microstructure and mechanical properties of this dual-phase HEA, *in situ* tensile experiments were conducted inside a TEM where unique plastic deformation behavior was captured at real time, as presented in Fig. 2 and Movie S1. With the increase of applied strain, dislocations were emitted from the phase boundary and then slipped into the FCC phase (marked with red dashed lines in Fig. 2a). However, dislocation activities were rarely observed in BCC phases even though the dislocations of the FCC phase were of dramatic interaction, suggesting a poor plasticity and high strength of the BCC phase. Moreover, multiple cross-slips of screw dislocations were activated by the accumulation of impressed strain at different sites within a planar slip band, shown by the white dashed line boxes in Fig. 2b. A zoom-in TEM image of the boxed region c (Fig. 2c) demonstrates that there were two active cross-slip systems coincided with different {111} compact planes, along which a full dislocation split into Shockley partials leaving stacking faults (SFs) in between. Figure 2d is the sequential snapshots of a dynamic cross-slip process, which illustrates that a perfect dislocation dissociated into two pairs of partials separated by SF3 and SF4, which annihilated at the free surface as a result of the subsequent movements of trailing partials.

Considering the much higher strength of BCC phase compared to that of FCC phase in the eutectic dual-phase AlCoCrFeNi HEA, the former served as the strengthening phase while the latter became the dominant carrier of deformation via dislocation plasticity, as evidenced by the *in-situ* deformation observations. At the early stage of deformation, plenty of dislocations were released from the phase boundary and glided along parallel slip bands, leading to the initial yielding. Despite of abundant dislocation sources, dislocation slips within the FCC phase became difficult due to the high lattice friction in HEAs^11^, the intrinsic strengthening effect of multiple solution atoms, and therefore cross slip were activated by homogenous increase of strain accumulation. The interaction between primary slip arrays and cross-slip systems was to increase the rate of dislocation storage by forming dislocation substructures, and to further promote work hardening of the FCC phase. Meanwhile, the BCC phase was difficult to deform even at crack tips with high stress, as evidenced by the *in situ* deformation experiments shown in Fig. S3. When the loading direction was perpendicular to the thickness direction of BCC lamellar, dense dislocations were blocked by the phase boundary, leading to the stress concentration, which made a small number of dislocations penetrate through or emitted from the boundary. But the glide of the passed dislocations was strongly hindered due to the high friction stress for motion of screw dislocations in BCC structure. Moreover, the ordered B2 structure can make the BCC phase much harder to deform plastically on account of more complex dislocation cores existing in ordered solutions^24^. Indeed, the plastic deformation of BCC phase was activated by the accumulation of applied stress but in a nonuniform manner, making confined contribution to the overall plasticity.

### The role of phase boundary in second phase strengthening

**Figure 3:**
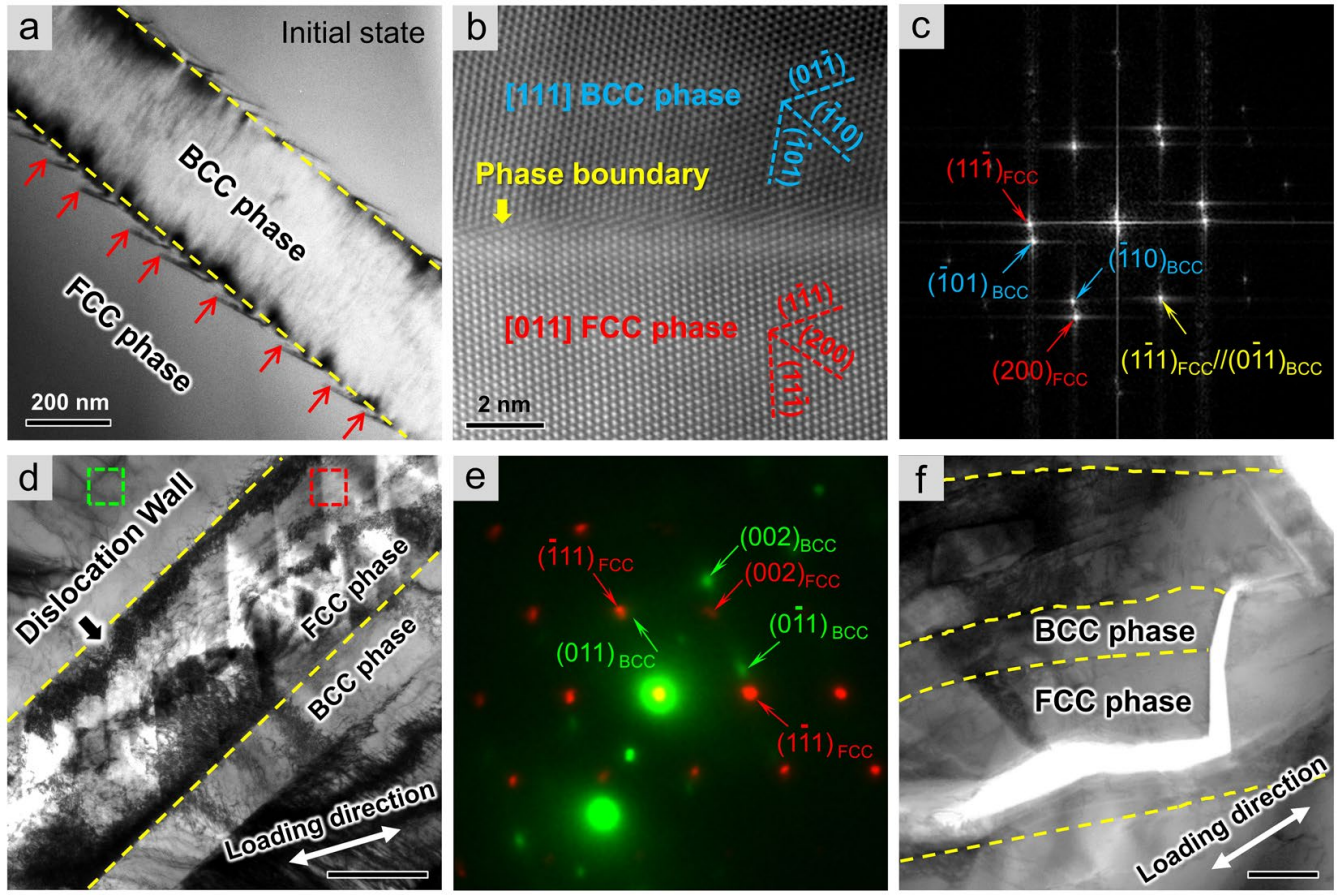
Phase boundary strengthening in the dual-phase AlCoCrFeNi_2.1_ HEA. (**a**) At the initial state, misfit dislocations were pinned at the phase boundaries with smooth morphology. (**b**,**c**) The phase boundary structure was shown by the HADDF-STEM image and corresponding FFT pattern, characterizing a semi-coherent interface with (1–11)_FCC_//(0–11)_BCC_. (**d**) Ultrahigh density of dislocations piled up at the phase boundary and strongly prevented the crack from expansion. (**e**) Overlapped diffraction patterns from boxed regions in (d) indicating another semi-coherent interface with (−111)_FCC_//(011)_BCC_. (**f**) The crack penetrated through the entire BCC phase immediately while the growth direction of crack was changed by dislocation motions in FCC counterparts.

As a result of further loading, dislocation slip was dramatically suppressed by the interaction among multiple dislocation arrays. In particular, dense dislocation walls formed along the phase boundaries within the FCC phase, indicating that the FCC and BCC phase boundaries acted as strong barriers for dislocation motion. After the initial stage of deformation when phase boundaries served as essential dislocation sources that triggered yield plasticity, phase boundary was found to play a significant role in preventing cracks from propagation, as shown in Fig. 3. Initially, the phase boundaries were straight and smooth with homogenous distribution of misfit dislocations (as shown in Fig. 3a), indicating the existence of semi-coherent interfaces. The orientation relationship between the neighboring two phases can be derived from the atomic configuration of semi-coherent phase boundary (Fig. 3b) and the corresponding fast Fourier Transform pattern (Fig. 3c), suggesting that (1–11)_FCC_//(01–1)_BCC_ and [011]_FCC_//[111]_BCC_, which coincides with the K-S orientation relationship. It is worth noting that a narrow band of high image intensity was clearly visible in the high-angle annular dark field (HAADF) scanning transmission electron microscopy (STEM) image (Fig. 3b), which marked a pronounced chemical concentration of heavy atoms at the interface of phase boundaries. Considering the physical nature of HEAs, the element segregation at phase boundaries should give rise to the formation of chemically disordered interfaces, which possessed higher strength compared with classical NiAl-based alloys^20^. Figure 3d shows a crack observed in an AlCoCrFeNi_2.1_ HEA subject to tensile deformation. In the direction of crack expansion, severe plastic deformation occurred in the local region ahead of the crack tip, where dislocations were piled up at the phase boundaries and obstructed crack extension by developing dense dislocation walls, resulting in a zigzag growth of the crack. The overlapped diffraction patterns captured within the boxed areas (as marked in Fig. 3d, the red one and the green one corresponding to the FCC and BCC phase respectively) were shown in Fig. 3e, indicating that the heavily deformed FCC phase was bounded by semi-coherent boundaries and the crystallographic orientation relationship between the FCC and adjacent BCC phase was close to (−111)_FCC_//(011)_BCC_ and [110]_FCC_//[100]_BCC_. However, in another case, when the loading geometry was changed to facilitate a crack propagation through the phase boundaries, the crack penetrated the phase boundaries via the cleavage of {110} planes with limited dislocation motion in the BCC phase while the extension of crack can transform to along another direction by activating cross-slip systems in the FCC counterpart (Fig. 3f), suggesting that the FCC phase was the paramount contributor to both the plasticity and damage tolerance of this eutectic dual-phase HEA.

It is a traditional method to strengthen alloys by introducing internal boundaries in order to impede the motion of dislocations. Unlike coherent twin boundaries with low excess energies, grain boundaries and phase boundaries are regarded as major strengthening agents for metallic materials because they are more effective in resisting dislocation penetration^14^. In this study, the phase boundaries were produced through the eutectic reaction during the preparation of dual-phase AlCoCrFeNi_2.1_ HEA. Thereby plenty of phase boundaries contained in this HEA were semi-coherent due to the low energy features^25^. Given that interfacial strength increases with the decrease of coherency, phase boundaries of semi-coherence prepared in eutectic dual-phase HEAs were observed to be strong enough to accommodate high stress due to their chemically disordered features, as proved by the HAADF-STEM observation (Fig. 3b) and *in-situ* straining experiments. Such strong phase boundaries were essential to achieving compatibility of interphase deformations through enhancing the strength of FCC phases by work hardening effects so as to commence dislocation glide in BCC phases.

### Phase strengthening vs. boundary strengthening in dual-phase high entropy alloys

**Figure 4:**
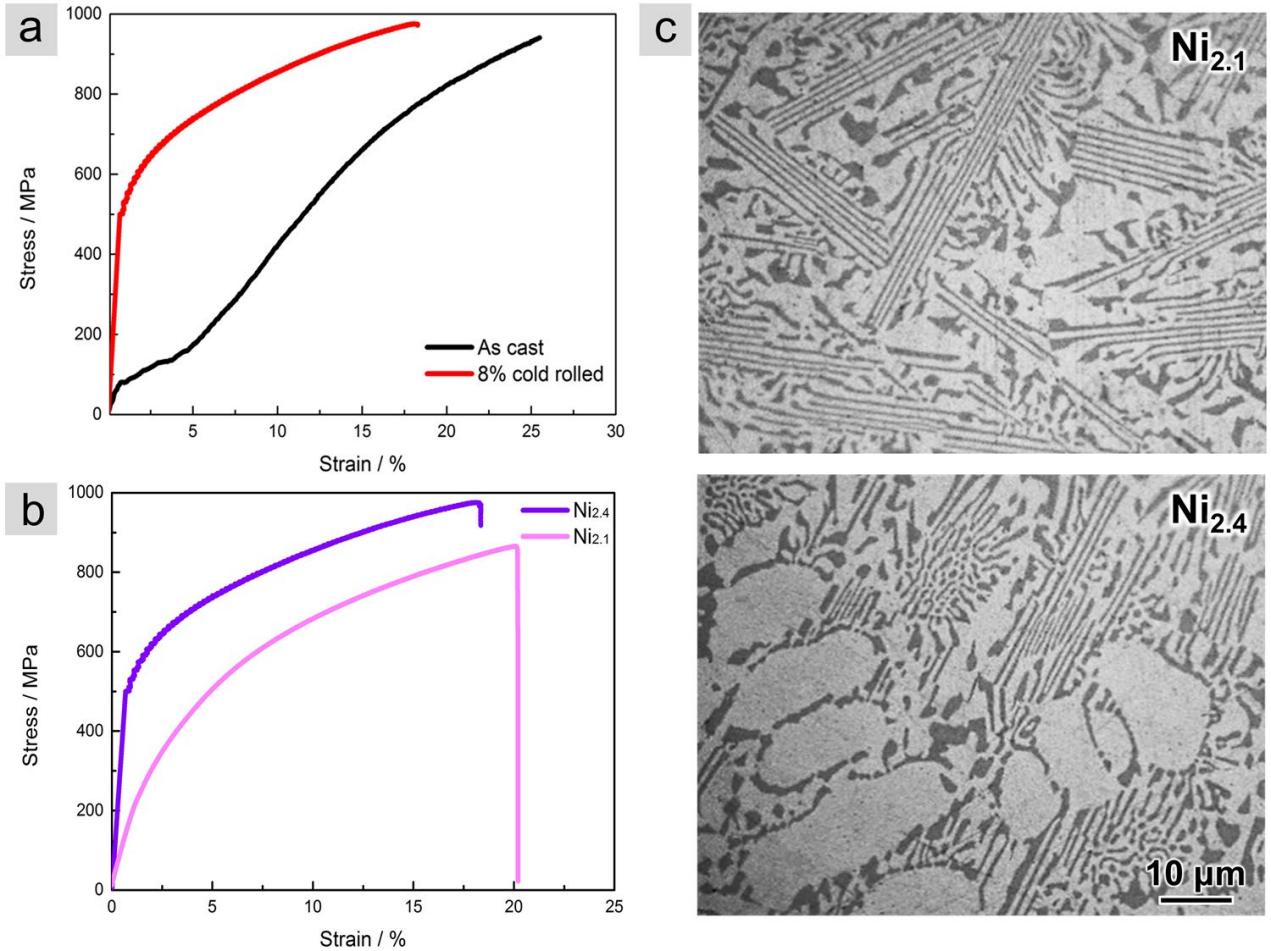
Mechanical property and microstructure of the eutectic dual-phase HEAs with different proportion of FCC phase. (**a**) Stress-strain curves of a dual-phase HEA (The dark line and the red line represented the as-cast sample and its cold-rolled counterpart respectively). (**b**) Stress-strain curves of two AlCoCrFeNi dual-phase HEAs distinguished by the proportion of FCC phase. (**c**) Ni_2.1_ (the pink line) and Ni_2.4_ (the purple line) corresponding to the one with less and more FCC phase respectively, as shown by the optical microscope images. The ones in deep color were BCC phases while the others were FCC phases.

Interestingly, the above results demonstrate that, in strong contrast to our traditional understanding, the FCC phase was capable of not only carrying large plastic deformation but also served as the source of strengthening. The high lattice friction in matrix and the strong semi-coherent phase boundaries which were decorated by disorderedly distributed multiple elements enabled high dislocation density in FCC phases. As a result, significant hardening of material would be expected. To confirm our hypothesis, we compared the mechanical properties of the as-casted and pre-strained AlCoCrFeNi alloys. The pre-straining was conducted to raise the dislocation density in the FCC phase. A comparison of the strain-stress curves of the as-casted and cold rolled dual-phase AlCoCrFeNi_2.1_ HEAs was shown in Fig. 4a. The strain-stress curve of the as-casted sample (black curve) exhibited a combination of high fracture strength (~0.94 GPa) and good ductility (~25%). Meanwhile, the mechanical property of the eutectic dual-phase HEA in cast can be further improved by reasonable pre-straining (shown by the red line in Fig. 4a), which remarkably increased the yield strength by a factor of ~5.

Moreover, increasing the proportion of pre-strained FCC phases may also raise the strength. The proportion of two phases can be tuned by the concentration of Ni. Figure 4b shows the stress-strain curves of two cold rolled eutectic dual-phase HEAs distinguished by the volume ratio of BCC/FCC, specifically 0.45 for the one named Ni_2.1_ and 0.3 for the other one named Ni_2.4_ (see optical microscope images in Fig. 4c). With the increase volume of BCC phase, the ultimate strength decreased from 0.97 GPa to 0.86 GPa, indicating an opposite relationship against the traditional principles^10^. This is due to the unique deformation mechanism observed in the eutectic dual-phase HEA where severe plastic deformation was constrained in the FCC phases to significantly strengthen FCC lamellas. However, it should be noted that a critical limit presumably exists in the proportion of two phases, beyond which the overall strength would decrease with more FCC phases due to the failure of second phase strengthening.

## Discussion

The properties of many metallic alloys are essentially related to the structure, morphology and chemical distribution of boundaries. In terms of atomic structure, the phase boundaries contained in eutectic dual-phase HEAs were supposed to be insufficient in prohibiting dislocations due to their semi-coherent nature, which is similar to those widely reported in NiAl dual-phase intermetallic alloys^26,27^. However, *in-situ* deformation tests revealed that dense dislocation storage can be achieved within FCC phases and dislocation walls were formed along phase boundaries. The unexpected strong interfaces are thought to be highly related to the chemically disordered distribution of multiple solution atoms at phase boundaries as well as in the matrix of HEAs^17^. Particularly, an ultra-thin segregation zone of randomly distributed Cr atoms, typically less than 2 nm in width (Fig. 3b and S4), was detected near the phase boundary without forming complex intermetallic compounds, which were intrinsically brittle and would lead to stress concentration^22^. Additionally, the solute segregation may enhance the thermodynamic stability and suppress diffusional boundary migration by lowering phase boundary energy, such that improving the mechanical properties of eutectic dual-phase HEAs at elevated temperature, which is consistent with the previous *ex-situ* results^20,28^. Beyond the effect of unique chemical constitution discussed above, cross slip of dislocations occurring in FCC phases also played a vital part in work hardening process by tuning dislocation interactions. Dislocation substructures, such as dense dislocation walls (Fig. 3d) and dislocation networks (Fig. S5), were developed at the final stage of uniform plastic deformation and dragged on crack propagation. Although BCC phases were strengthening components in the eutectic dual-phase HEA due to their intrinsically large lattice resistance, which made them hard to deform plastically, the existence of semi-coherent phase boundaries played an essential part in balancing strength-ductility. Considering the brittleness of ordered BCC phases, the unexpected strong phase boundaries can prevent massive dislocations from penetrating, thus not only reducing dislocation pile-ups in BCC phases, which may facilitate the nucleation of cracks, but also promoting work hardening process in FCC phases by achieving dense dislocation storage.

In conclusion, by conducting *in situ* deformation experiments in a eutectic dual-phase AlCoCrFeNi HEA, a unique mechanism of second phase strengthening was revealed. In contrast to the traditional thought, which considers hard BCC phases as the crucial strengthening component in dual-phase alloys, it is found that pre-strained FCC can also make significant contribution to the macroscopic strength of the eutectic dual-phase AlCoCrFeNi HEA. Consequently, increasing the proportion of pre-strained FCC phases can effectively enhance the strength of material. The strengthening effect of this eutectic dual-phase HEAs lies in several aspects. Firstly, given the large lattice resistance to dislocation gliding, BCC phases served as powerful barriers to prevent massive dislocations from moving inside, which intrinsically enhanced the overall strength. Moreover, the unexpected strong phase boundaries made great contribution in balancing strength-ductility while the abundant cross-slip activities can increase the rate of dislocation storage and made FCC phases exceptionally strong. Our results not only revealed the underlying strengthening mechanism of FCC/BCC dual-phase HEAs, but also shed light on new strategies for optimizing the mechanical properties of dual-phase HEAs.

## Methods

Commercially pure elements Al, Co, Ni, Cr (99.9 wt. %) and Fe (99.6 wt. %) were alloyed by vacuum arc melting to prepare the dual-phase high entropy alloy of AlCoCrFeNi_2.1_ and AlCoCrFeNi_2.4_. It is worth to note that the AlCoCrFeNi HEAs tested in our study were designed following the concept of eutectic reaction, and the accurate eutectic composition was found to be AlCoCrFeNi_2.1_ while the melting temperature was set to be 1773 K, as evidenced by the differential scanning calorimetric (DSC) analysis^20^. The alloys were repeated melting at least five times with electromagnetic stirring for homogeneity. Then the alloys were directly solidified in a water-cooled cold copper hearth and the solidified ingots were about 30 mm in diameter and 15 mm in thickness. The *in situ* tensile test was conducted by a Gatan 654 single-tilt straining holder at a typical strain rate of ~1.0 μm∙s^−1^ inside an FEI Tecnai G2 F20 S-TWIN TEM with accelerating voltage of 200 kV. An FEI Titan G2 80–200 ChemiSTEM, equipped with Cs corrector and operated at 200 kV, was also used to characterize the atomic level structure and elements distribution. In a representative process, the sample for *in situ* tensile test was cut into 3 × 3 × 1 mm square pieces by wire cut electrical discharge machining and then polished down to ~40 μm in thickness before producing electron transparent regions for TEM observation using ion milling. Room-temperature tensile tests were performed using the Instron 5569 testing machine at a constant strain rate of 1 × 10^−3^ s^−1^ and the tensile test specimens cut from as-cast alloys had a diameter of 8 mm and gauge length of 40 mm. Phase analyses (microstructure and element constituents) of Ni_2.1_ and Ni_2.4_ alloys were conducted by a Zeiss Axio Imager 2 polarizing microscope and a Hitachi SU-70 SEM operating at 30 kV after sequentially polished and etched the alloys with the 10 vol. % aqua regia-90 vol. % ethanol solution at room temperature.

## Electronic supplementary material

Supplementary Information

Movie S1

## Data Availability

All data needed to evaluate the conclusions in the paper are present in the paper and/or the Supplementary Information. Additional data related to this paper may be requested from Q.Y. (qian_yu@zju.edu.cn).
