# Supplementary material for: The Exceptional Strong Face-centered Cubic Phase and Semi-coherent Phase Boundary in a Eutectic Dual-phase High Entropy Alloy AlCoCrFeNi
Source: Sci Rep. 2018 Oct 8;8:14910. doi: 10.1038/s41598-018-33330-0 (PMC6175879; doi:10.1038/s41598-018-33330-0)
Supplement: Supplementary file 1 — Supplementary Information [file 41598_2018_33330_MOESM1_ESM.pdf]

Supplementary Information for

The Exceptional Strong Face-centered Cubic Phase and Semi-coherent Phase Boundary in a Eutectic Dual-phase High Entropy Alloy AlCoCrFeNi

Qiannan Wang, Yiping Lu, Qian Yu, Ze Zhang

This PDF file includes:

- Figure S1 Characterization of lamellar FCC/BCC phases at multiple scales.
- Figure S2 Original EDS mapping results shown in Fig. 1b
- Figure S3 Dislocation activities in BCC phase at high stress state.
- Figure S4. Atomic-scale EDS mapping indicating distinct enrichment of Cr at the phase boundary.
- Figure S5 Dislocation networks formed at the phase boundary.
- Table S1 Chemical composition of FCC and BCC phases in $AlCoCrFeNi_{2.1}$ and $AlCoCrFeNi_{2.4}$ high-entropy alloys.
- Legends for Movie captions

Other Supplementary Material for this manuscript includes the following:

- Movie S1 Dislocation cross-slip process in the FCC phases.

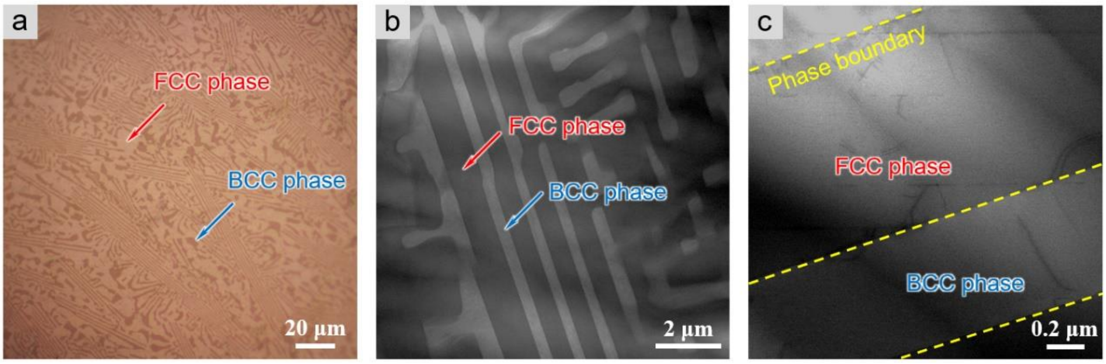

Figure S1 Characterization of lamellar FCC/BCC phases at multiple scales. (**a**) The optical micrograph of eutectic AlCoCrFeNi2.1 HEA, the dark and light brown one corresponding to BCC and FCC phase respectively. (**b-c**) TEM images captured with different magnifications.

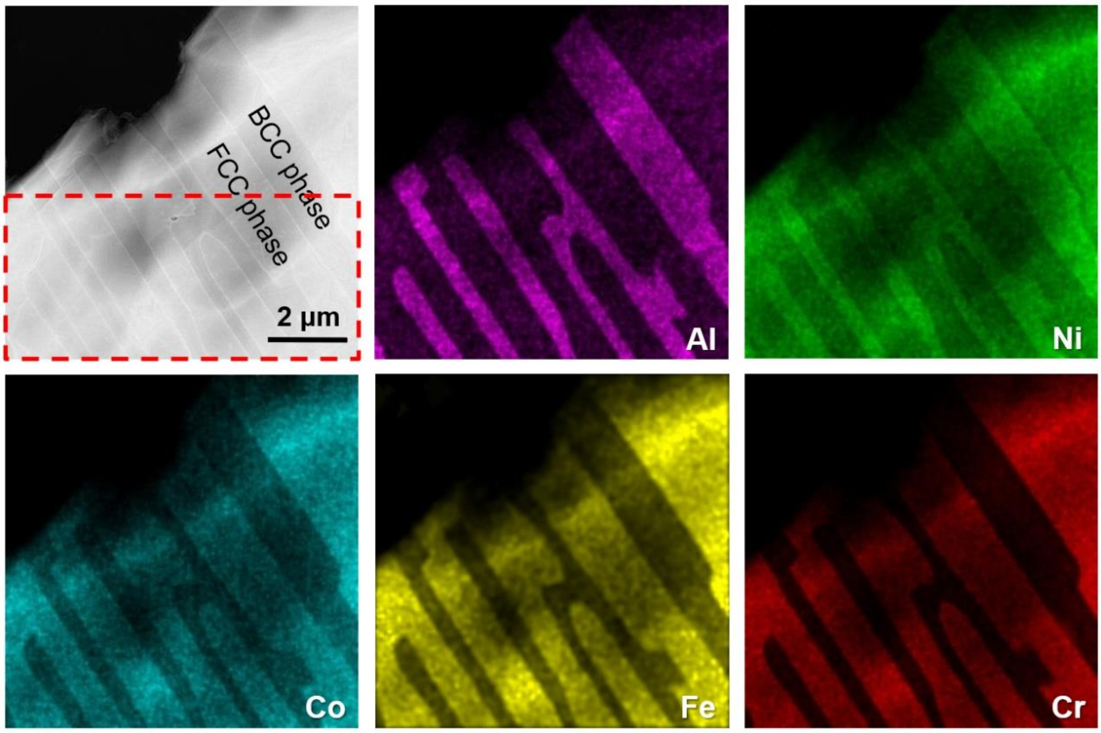

Figure S2 Original EDS mapping results shown in Fig. 1b. The region marked by red dashed lines was presented in Fig. 2b to illustrate the distribution of chemical elements in the dual phase AlCoCrFeNi2.1 HEA.

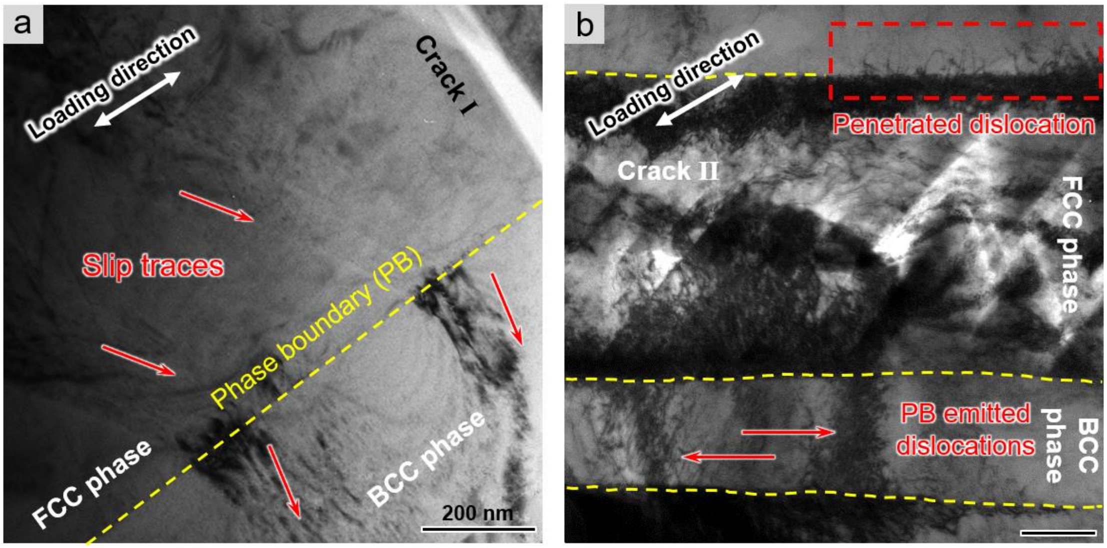

Figure S3 Dislocation activities in BCC phase at high stress state. (**a**) With the increase of applied stress, some dislocations penetrated the phase boundary and then blocked in the BCC counterpart due to its high lattice resistance to dislocation motion. (**b**) As a result of work hardening, massive dislocations piled up at the phase boundaries in the FCC phase while a small number of dislocations passing through or emitted from phase boundaries.

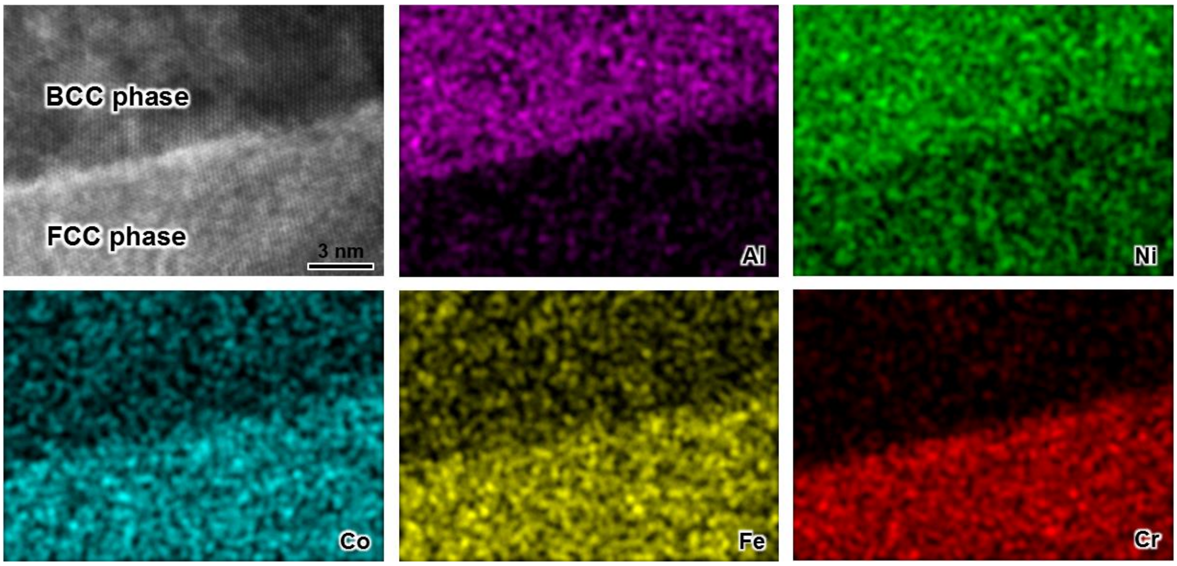

Figure S4. Atomic-scale EDS mapping indicating distinct enrichment of Cr at the phase boundary. Common but differentiated distribution of elements was characterized by EDS mapping, indicating distinct enrichment of Cr was concentrated at the phase boundary.

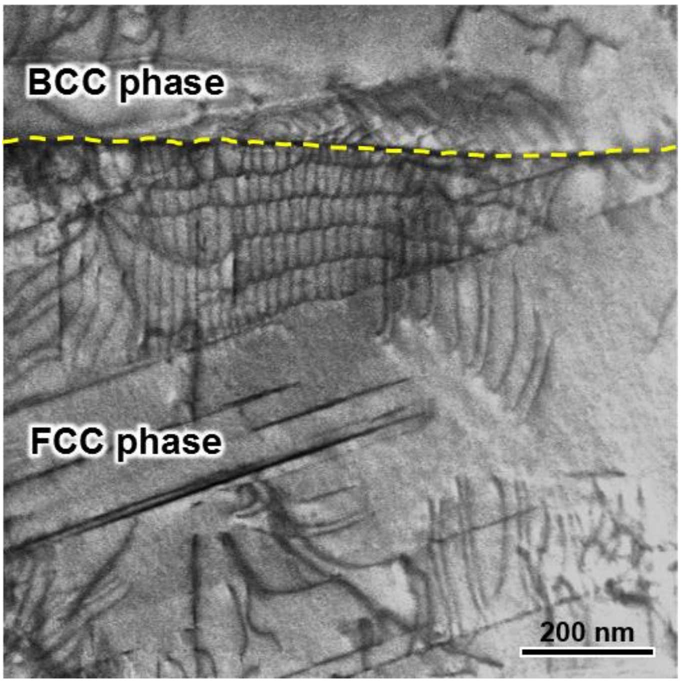

Figure S5. Dislocation networks formed at the phase boundary. A dislocation network formed at the phase boundary as a result of dislocation interactions.

Table S1 Chemical composition of FCC and BCC phases in $AlCoCrFeNi_{2.1}$ and $AlCoCrFeNi_{2.4}$ high-entropy alloys.

Alloy	Phase	Chemical composition / at. %				
		Al	Co	Cr	Fe	Ni
$AlCoCrFeNi_{2.1}$	FCC	11.87	17.79	19.81	18.90	31.63
	BCC	29.22	12.70	8.95	10.96	38.17
$AlCoCrFeNi_{2.4}$	FCC	11.76	16.22	19.13	17.07	35.82
	BCC	29.07	11.22	8.41	9.74	41.56

Movie Captions:

Movie S1 Dislocation cross-slip process in the FCC phases. Dynamic process of cross slip observed in the FCC phase illustrated that a full dislocation dissociated into partials and then annihilated at the surface. The movie was speeded up for 10 times.
